# Leucyl-tRNA Synthetase Regulates Lactation and Cell Proliferation via mTOR Signaling in Dairy Cow Mammary Epithelial Cells

**DOI:** 10.3390/ijms15045952

**Published:** 2014-04-09

**Authors:** Lina Wang, Ye Lin, Yanjie Bian, Lili Liu, Li Shao, Lin Lin, Bo Qu, Feng Zhao, Xuejun Gao, Qingzhang Li

**Affiliations:** Key Laboratory of Dairy Science of Ministry of Education, Northeast Agricultural University, Harbin 150030, China; E-Mails: wanglina_6688@126.com (L.W.); linlu516@163.com (Y.L.); bianjie.033@163.com (Y.B.); liulili-liulili@163.com (L.Liu); shao880330@126.com (L.S.); lin211314.love@163.com (L.Lin); qb5172@163.com (B.Q.); erjinzhi@126.com (F.Z.); gaoxj5390@sina.com (X.G.)

**Keywords:** dairy cow mammary epithelial cells, lactation, leucine, cell proliferation, LeuRS, mTOR

## Abstract

The role of LeuRS, an aminoacyl-tRNA synthetase, as an intracellular l-leucine sensor for the mTORC1 pathway has been the subject of much research recently. Despite this, the association between LeuRS and lactation in dairy cow mammary epithelial cells (DCMECs) remains unknown. In this study, we found that LeuRS expression in mammary gland tissue was significantly higher during lactation than pregnancy. Moreover, our data demonstrates that LeuRS is localized in the cytoplasm. Treatment with leucine increased DCMECs viability and proliferation, as well as mammalian target of rapamycin (mTOR), p-mTOR, ribosomal protein S6 kinase 1 (S6K1), p-S6K1, β-Casein, sterol regulatory element binding protein 1c (SREBP-1c), glucose transporter 1 (GLUT1), and Cyclin D1 mRNA and protein expression. Secretion of lactose and triglyceride were also increased. siRNA-mediated knockdown of LeuRS led to reduction in all of these processes. Based on these data, LeuRS up-regulates the mTOR pathway to promote proliferation and lactation of DCMECs in response to changes in the intracellular leucine concentration.

## 1. Introduction

Lactation regulation has been the subject of much scientific and clinical interest for several decades. More specifically, the effect of hormones and amino acids on lactation in the dairy cow has been studied since the last century [1–3]. Leucine and other essential amino acids have been linked to processes performed by the mammary gland of dairy cows [4]. Combined with these insights, researchers began to focus on the molecular regulation mechanisms of amino acids on lactation in the last decade [5–7]. mTOR is a serine/threonine kinase that integrates signals from amino acids, growth factors, and the energy status of the cell. This signaling molecule plays an important role in a multitude of cellular processes, including cell growth and protein translation [8,9]. In recent years, studies have focused on the nutrient regulation of mTORC1, one of the two complexes of mTOR [10–12]. Leucine is currently the most prevalent essential amino acid in the diet. Present as a branched chain amino acid, leucine is reportedly involved in the regulation of cell growth, proliferation, and differentiation through mTOR [13–15]. Moreover, leucine restriction produced dose-dependent regulation of mTOR in ATCD5 cell [16].

The precise mechanism of how mTORC1 activation is mediated by intracellular leucine sensing is poorly understood. Several studies have identified LeuRS as a key intracellular regulatory factor of the mTORC1 pathway in 293T cells [17–19]. However, the mechanism of how LeuRS senses to leucine and acts on mTORC1 to regulate lactation is unknown in DCMECs. In this study, we demonstrate that LeuRS plays a critical role in milk synthesis by sensing the intracellular leucine concentration and regulating amino acid-induced mTORC1 activation.

## 2. Results and Discussion

### 2.1. Localization and Expression of LeuRS in Mammary Gland Tissues

**Figure 1.**
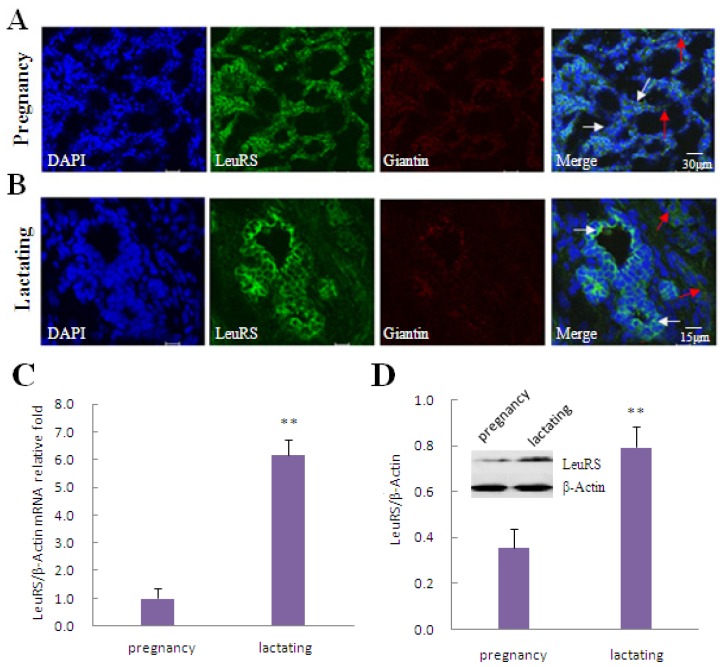
Localization and expression of LeuRS in mammary gland tissue and DCMECs. (**A**,**B**). Localization of endogenous LeuRS in pregnancy (**A**) and lactating (**B**) mammary glands. LeuRS were counterstained with FITC, giantin as a protein localized in the Golgi apparatus were counterstained with Cy5, and nuclei were counterstained with 4′,6-diamidino-2-phenylindole (DAPI). Red arrows indicated LeuRS expressed in the connective tissues while white arrows designated LeuRS expressed in the acinar buds. Scale bars: 30 and 15 μm, respectively. Representative images from one of three or more independent experiments; (**C**) Relative levels of LeuRS mRNA in pregnancy and lactating mammary gland tissues as determined by qRT-PCR; (**D**) Levels of LeuRS protein in pregnancy and lactating mammary gland tissues as determined by western blotting analysis. β-Actin was assessed as a loading control. A representative blot and quantitation of three independent experiments were shown. In all panels, data represent the mean ± SD of three independent experiments. ^**^ *p* < 0.01.

In this study, LeuRS was expressed in both pregnancy and lactating mammary glands in Holstein dairy cows. In pregnancy mammary gland tissue LeuRS showed no difference in the acinar buds (Figure 1A, white arrows) and the connective tissue (Figure 1A, red arrows). While in lactating mammary gland tissue, most of LeuRS was expressed in the acinar buds (Figure 1B, white arrows) with only a small amount detected in the connective tissue (Figure 1B, red arrows). Quantitative real-time PCR (qRT-PCR) (Figure 1C) and western blotting analysis (Figure 1D) revealed that the level of LeuRS expression was significantly higher in lactating mammary gland tissue than in pregnancy mammary gland tissue.

### 2.2. Culture and Identification of DCMECs and Cytolocalization of LeuRS

**Figure 2.**
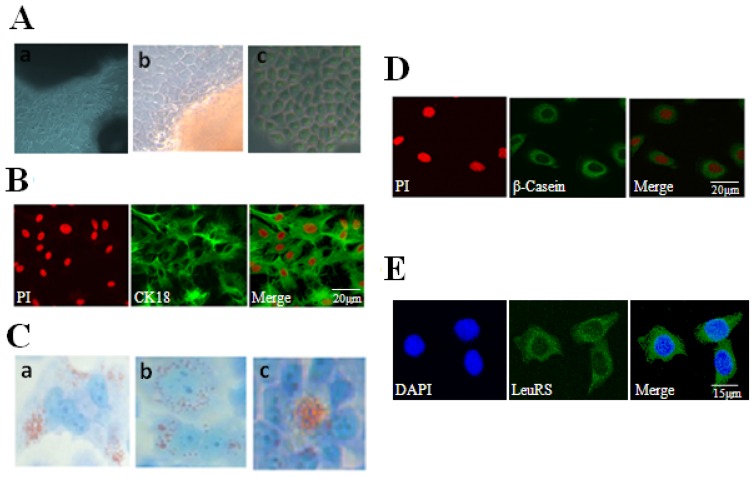
Culture and identification of DCMECs and cytolocalization of LeuRS. (**A**) Culture and purification of DCMECs. (**a**) A large number of fibroblasts and a small amount of mammary epithelial cells were cultured and then observed by contrast phase microscopy (200×); (**b**) Mammary epithelial cells arose from seeded tissues, as observed by contrast phase microscope (400×); (**c**) Cellular morphology of purified DCMECs was observed by contrast phase microscope (400×); (**B**) Purified DCMECs expressed CK18. CK18 was counterstained with FITC, and nuclei were counterstained with propidium iodide (PI). Scale bar: 20 μm; (**C**) Detection of lipid droplets in DCMECs with Oil Red O. Lipid droplets were special with red (400×). (**a**) Lipid droplets were localized to one side of the cytoplasm; (**b**) Lipid droplets distributed throughout the cytoplasm evenly; (**c**) Lipid droplets were secreted to the acinar cavity; (**D**) Detection of β-Casein in DCMECs. β-Casein was counterstained with FITC, and nuclei were counterstained with PI. Scale bar: 20 μm; (**E**) Localization of endogenous LeuRS in lactating DCMECs. LeuRS was counterstained with FITC, and nuclei were counterstained with DAPI. Scale bar: 15 μm. Representative images from one of three independent experiments.

The primary cells were seeded from mammary tissues *in vitro* and cultured for five to ten days to produce a mixed culture of fibroblasts and a small number of mammary epithelial cells (Figure 2Aa). In addition, mammary epithelial cells arose from some seeded tissues (Figure 2Ab). Because fibroblasts and mammary epithelial cells have different sensitivity to trypsin, DCMECs were purified by digesting with 0.25% trypsin 3–5 times to remove fibroblasts. Contrast phase microscopy revealed that these purified DCMECs exhibit a flattened morphology with a beehive-shaped arrangement as expected (Figure 2Ac). These cells also stained positively for the epithelial cell marker cytokeratin 18 (CK18) (Figure 2B). In addition, we could visualize lipid droplets with Oil Red O staining (Figure 2C). Interestingly, β-Casein could also be detected in DCMECs, suggesting that purified DCMECs may function in secreting lactoprotein (Figure 2D). After cytolocalization of LeuRS, we found that LeuRS was expressed and localized in the cytoplasm of DCMECs (Figure 2E).

### 2.3. The Effect of Leucine on LeuRS to Regulate Cell Growth and Proteins in mTOR Signaling in DCMECs

**Figure 3.**
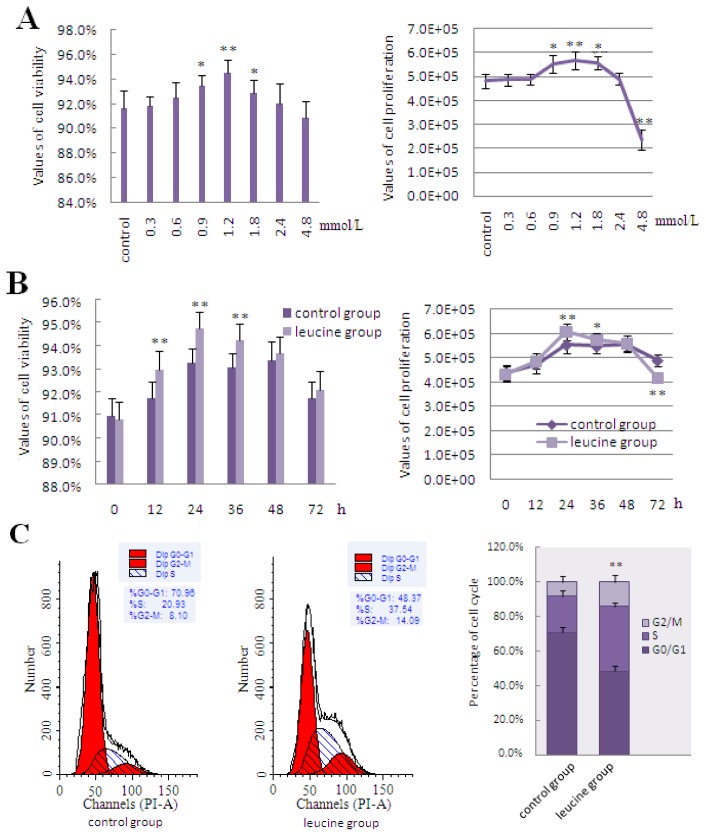

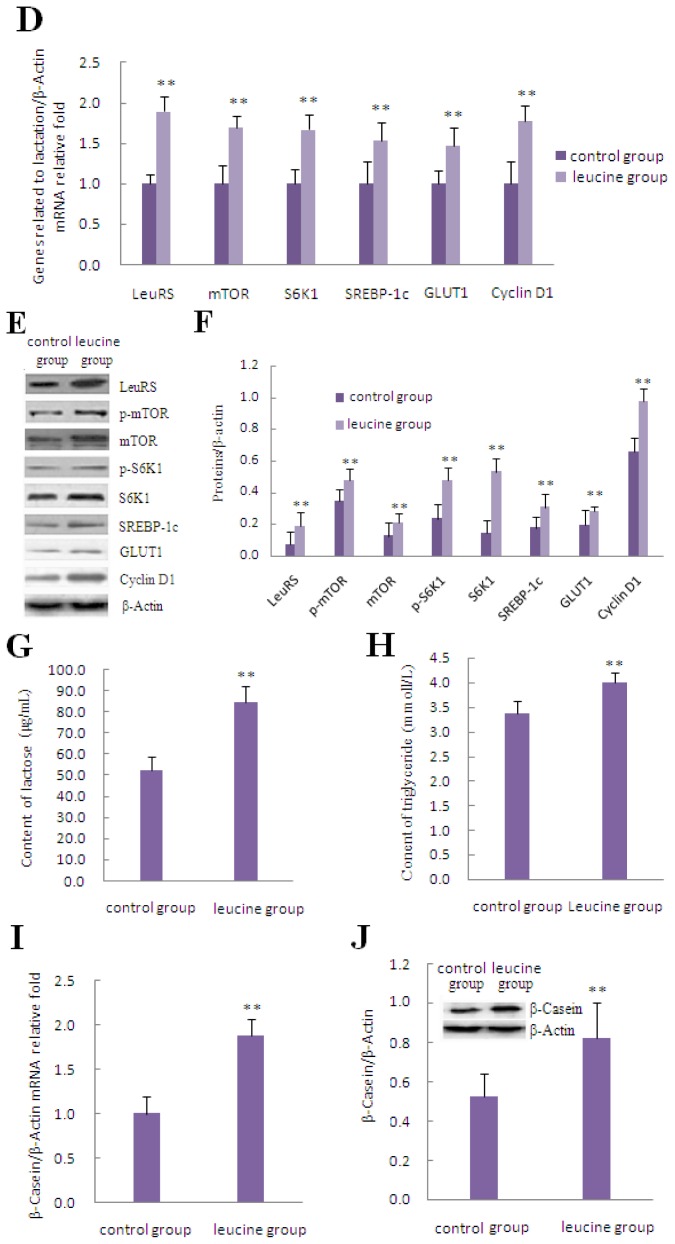
The effect of leucine on LeuRS to regulate cell growth and expression of proteins involved in mTOR signaling in DCMECs. (**A**) DCMECs were left untreated (control) or treated with varying concentrations of leucine (0.3, 0.6, 0.9, 1.2, 1.8, 2.4, and 4.8 mmol/L) for 24 h; (**B**) DCMECs were treated with 1.2 mmol/L leucine for the times indicated, and then cell viability and proliferation were determined by using the CASY-TT assay; (**C**) Examination of cell cycle progression in DCMECs treated with 1.2 mmol/L leucine for 24 h. Cells were stained with PI and then analyzed by flow cytometry. Representative histograms and quantitation of three independent experiments were shown; (**D**) Changes in the level of lactation- and mTOR signaling-related gene expression in response to 1.2 mmol/L leucine treatment for 24 h as determined by qRT-PCR; (**E**) Representative western blotting of mentioned proteins. DCMECs were either left untreated (control) or treated with 1.2 mmol/L leucine for 24 h. β-Actin was assessed as a loading control; (**F**) Quantitation of (**E**); (**G**,**H**) Secretion of lactose and triglyceride into the culture medium were detected by ELISA in DCMECs treated with 1.2 mmol/L leucine for 48 h; (**I**) β-Casein mRNA expression in DCMECs treated with 1.2 mmol/L leucine for 24 h as measured by qRT-PCR; (**J**) Level of β-Casein protein in DCMECs treated with leucine for 24 h as assessed by western blotting analysis. A representative blot and quantitation of three independent experiments were shown. In all panels, data represent the mean ± SD of three independent experiments, and three or six wells per treatment within each independent experiment. ^*^ and ^**^ indicate *p* < 0.05 and *p* < 0.01, respectively.

The effects of leucine on cell viability and cell proliferation were both dose- and time-dependent. Treatment of DCMECs with increasing doses of leucine increased cell viability and cell proliferation, both of which peaked at 1.2 mmol/L. While cell viability and cell proliferation increased with further increases in leucine concentration to 1.8 and 2.4 mmol/L, the increments of addition were less than that observed at 1.2 mmol/L. However, when the extracellular leucine concentration reached 4.8 mmol/L, DCMECs were unable to generate enough energy to actively transport the extra leucine residues through the leucine transporter. Excess leucine DCMECs resulted in metabolic acidosis, thus worsening cell state and reducing cell viability and proliferation compared with the control group (Figure 3A). Leucine-mediated cell viability was increased after incubation with 1.2 mmol/L leucine for 12, 24, and 36 h, while the greatest induction of cell proliferation was observed at 24 h of incubation with leucine. DCMECs reached the logarithmic growth phase, which was the most productive stage of growth and allowed maximum nutrient absorption and utilization efficiency in the medium, 24 h after seeding in culture plates. Then at 36 and 48 h, DCMECs were found to be in the stationary phase, where there was little change in the rates of cell viability and proliferation. At about 72 h, DCMECs began to enter a decline phase due to contact-inhibition of cell proliferation, as well as the lack of nutrients and accumulation of metabolites in the culture medium, therefore, the cell viability and cell proliferation decreased significantly compared with the previous stage of growth (Figure 3B). Flow cytometric analysis of cell cycle progression demonstrated that treatment with leucine increased the frequency of cells in the S and G2/M phases by 16.61% and 5.99%, respectively, further suggesting that leucine stimulated cell proliferation (Figure 3C). Treatment with leucine resulted in markedly increased expression of lactation- and mTOR signaling-related molecules, including LeuRS, mTOR, S6K1, SREBP-1c, GLUT1, and Cyclin D1, compared with the control group (Figure 3D–F). Meanwhile, the secretion of lactose and triglyceride, as well as β-Casein expression, increased significantly after treatment with leucine (Figure 3G–J).

### 2.4. LeuRS Knockdown Reduced Cell Growth, the Expression of Lactation-Associated Proteins, and Milk Synthesis

**Figure 4.**
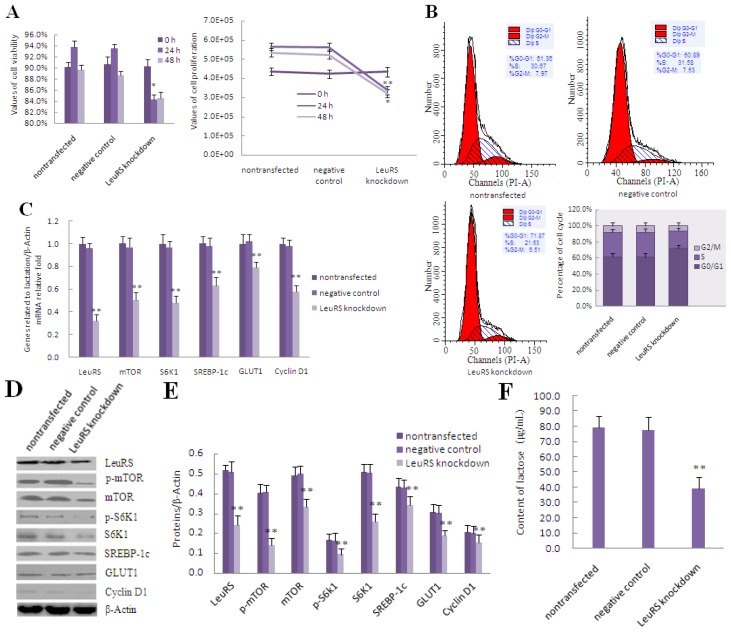

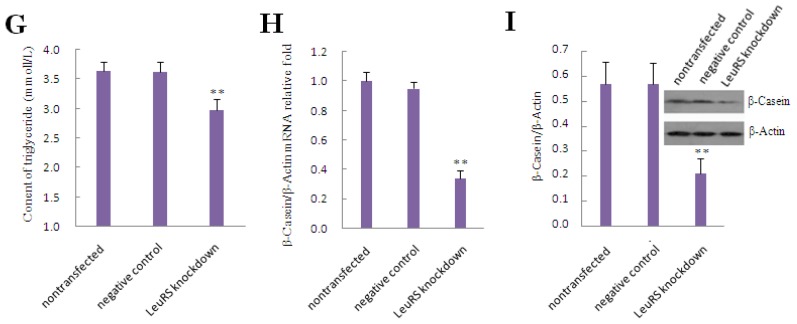
The effect of LeuRS knockdown on leucine-induced proliferation and expression of lactation-associated proteins in DCMECs. DCMECs were divided into three groups: nontransfected group without siRNA and Lipofectamine™ 2000 reagent, control group with a negative siRNA and Lipofectamine™ 2000 reagent as well as transfected group with a LeuRS-specific siRNA and Lipofectamine™ 2000 reagent, all groups were treated with 1.2 mmol/L leucine. (**A**) Cell viability and cell proliferation after LeuRS knockdown for 0, 24 and 48 h were measured by using CASY-TT; (**B**) Measurement of the cell cycle progression by flow cytometric analysis of PI-stained DCMECs after LeuRS knockdown for 24 h; (**C**) Changes in the level of lactation- and mTOR signaling-related gene expression in response to LeuRS knockdown for 24 h as measured by using qRT-PCR; (**D**) Representative western blotting of mentioned proteins. DCMECs were treated as mentioned above for 24 h. β-Actin was assessed as a loading control; (**E**) Quantitation of (**D**); (**F**,**G**) Lactose and triglyceride secreted into the culture medium were detected after LeuRS knockdown for 48 h; (**H**) β-Casein expression in DCMECs was detected by using qRT-PCR after LeuRS knockdown for 24 h; (**I**) Level of β-Casein protein in DCMECs after LeuRS knockdown for 24 h as assessed by western blotting analysis. β-Actin was assessed as a loading control. A representative blot and quantitation of three independent experiments are shown. In all panels, data represent the mean ± SD of three independent experiments, and three or six wells per treatment within each independent experiment. ^*^ and ^**^ indicate *p* < 0.05 and *p* < 0.01, respectively.

To evaluate whether LeuRS is associated with the regulation of lactation, we employed siRNA to knockdown LeuRS expression and treated DCMECs with leucine for 0, 24 and 48 h. Our data demonstrated that cell viability and cell proliferation were reduced after LeuRS knockdown in leucine-induced DCMECs compared with the negative control group (Figure 4A). Flow cytometric analysis revealed that the respective frequency of S and G2/M phases decreased 9.95% and 1.02% compared with the negative control group (Figure 4B). The levels of mTOR, S6K1, SREBP-1c, GLUT1, and Cyclin D1 were also reduced following LeuRS knockdown (Figure 4C–E). Additionally, secretion of lactose and triglyceride, along with β-Casein expression were all reduced (Figure 4F–I), suggesting that milk lactation in LeuRS-deficient DCMECs treated with leucine was reduced dramatically.

### 2.5. Discussion

This is the first study investigating the role of LeuRS, an enzyme expressed in the mammary gland of Holstein dairy cows during pregnancy and lactation. Expression of LeuRS during lactation was significantly higher than during pregnancy, which was consistent with mammary gland development and the biological function of LeuRS. On one hand, during lactation, acinar development is mature and lactoprotein synthesis in acinar epithelial cells is increased dramatically [20]. On the other hand, LeuRS is an aminoacyl-tRNA synthetase that specifically catalyzes the combination of tRNA molecules with leucine residues. In addition, LeuRS expression increases concurrently with the sharp increase in lactoprotein during lactation [21]. We found that LeuRS was expressed and localized in the cytoplasm of DCMECs.

In this study, we purified mammary epithelial cells from Holstein dairy cows for use in experimental assays. These DCMECs exhibited the typical cobblestone-like morphology that differed significantly from fibroblasts; we also found the expression of the epithelial cell-specific keratin CK18. Furthermore, analysis of the lactating ability of DCMECs revealed that these DCMECs can synthesize β-Casein, which was localized in the cytoplasm. Additionally, lipid droplets secreted from the cytoplasm were observed through staining with Oil Red O, an azo dye that is known to preferentially stain fat [22]. Interestingly, we found three types of lipid droplets secreted by DCMECs. Some lipid droplets were localized to one side of the cytoplasm, while some were distributed throughout the cytoplasm evenly. Others were secreted to the acinar cavity. The presence of all these kinds of lipid droplets demonstrated that DCMECs exhibited a specific phenotype during lactation.

We tested the effects of leucine on the proliferative and secretory properties of DCMECs. Our results showed that proteins related to proliferation and lactation were increased in DCMECs treated with 1.2 mmol/L leucine for 24 and 48 h. More recent studies on the relationship between leucine and mTOR demonstrate that this amino acid is not only a substrate for protein synthesis, but also is an important regulatory factor in the control of mTOR signaling [14,23,24]. Kimball *et al*. hypothesized that essential amino acids may exist at the center of mTOR signaling [25]. Amino acid availability, in particular the availability of the essential amino acid leucine, is required for mTORC1 activation [26]. Nicklin *et al*. proposed an important model for the molecular mechanism of leucine signaling to mTORC1. When cells are starved for glutamine, leucine cannot be transported into cells and mTORC1 is not activated. Intracellular leucine is sensed by an unknown factor that activates mTORC1 through Rag GTPases or an unknown pathway [27]. Although a few amino acid transporters have been identified, the identity and location of the intracellular amino acid sensor involved in regulating mTORC1 has remained elusive [28].

Moreover, the mechanism underlying leucine-mediated activation of mTORC1 remains controversial. Recently, the class III PI3K, vacuolar protein sorting 34 (Vps34), was proposed to be activated by amino acid and involved in the transduction of signals from amino acids to mTORC1 [29–31]. Most recent studies in mammalian cells and *Drosophila* identified Rag GTPases as activators of mTORC1 that sense amino acids signals [32–34]. These different hypotheses reflect the complexity of signaling from amino acids, particularly leucine to mTORC1 activation. Nevertheless, additional studies are required to understand how mTORC1 responds to amino acids.

The molecular mechanism of leucine-mediated control of lactoprotein synthesis remains poorly understood [35,36]. Our data suggest that leucine concentration is sensed by LeuRS, which transmits signals from the amino acid to mTORC1 in DCMECs. Leucine can up-regulate the expression of mTOR and S6K1 in amino acid-mTORC1 signaling pathway sensed by LeuRS. In addition, leucine plays a role as a positive regulator of mTOR, SREBP-1c, GLUT1, Cyclin D1 and β-Casein expression. Leucine also plays an important regulatory role in lactation mediated by LeuRS.

To address the mechanism of leucine activation of mTORC1, we investigated the effect of LeuRS knockdown on DCMECs proliferation and protein expression. Our data demonstrate that siRNA-mediated silencing of LeuRS decreased cell proliferation and the expression of lactation- and mTOR signaling-related proteins, suggesting that LeuRS is a regulatory factor involved in the amino acid/mTOR signaling pathway in DCMECs. Moreover, LeuRS functions as a GTPase-activating protein (GAP) for Rag GTPase to activate mTORC1, indicating that LeuRS is a key mediator of amino acid signaling to mTORC1 in 293T cells [19]. The amino acid-dependent shuttling of mTOR requires Rag GTPases and a trimeric protein complex known as the regulator [11,33]. Other intracellular proteins have also been implicated in amino acid sensing, including Vps34, which regulates shuttling events in the lysosome system [30,37,38]. Based on these findings, LeuRS plays a critical role in amino acid-induced mTORC1 activation by sensing the intracellular leucine concentration and initiating the molecular events leading to mTORC1 activation. However, further investigation is required to determine whether LeuRS binds directly to Rag GTPase and how LeuRS regulates the GTP/GDP cycling of Rag heterodimers in mTORC1 activation in DCMECs. Additionally, studies are needed to elucidate whether a relationship exists between LeuRS and Vps34. This is the first report that LeuRS promotes lactation-related gene expression in DCMECs via the mTOR pathway.

The ability of mammary glands to produce milk is determined by the number of cells and their level of viability [39]. The main component of sugar—lactose, the main component of milk fat—triglyceride and a major lactoprotein—β-Casein, also demonstrate the secretary ability of DCMECs [40,41]. Therefore, assessing cell proliferation and secretion of lactose, triglyceride, and β-Casein are meaningful parameters for studying lactation. Our results revealed that treatment of DCMECs with 1.2 mmol/L leucine increased SREBP-1c, Cyclin D1, β-Casein, triglyceride, and lactose. CASY-TT and flow cytometric analysis demonstrated that leucine promotes proliferation, posssibly by regulating Cyclin D1 or an mTOR-independent mechanism. Consistent with this, siRNA-mediated knockdown of LeuRS down-regulated the expression of these genes even in the presence of 1.2 mmol/L leucine. Our results also demonstrate that leucine may up-regulate GLUT1 expression and lactose production. However, this result contrasts markedly with several previously published studies. Nishitani *et al*. proposed that leucine stimulates glucose transport in skeletal muscle via the PI3-kinase and PKC pathways independent of mTOR and insulin [42]. However, Burnol *et al*. reported that to a certain degree, amino acid availability had no effect on lactose secretion [43,44]. It is unclear whether the regulation between LeuRS and GLUT1 is direct or through expressions of key genes involved in synthesis, such as mTOR and IRS among others. SREBP-1c, which is downstream of mTORC1, is a key regulator of fat synthesis in the dairy cow [45–47]. Thus, leucine or LeuRS may mediate mTOR signaling to regulate SREBP-1c and triglyceride secretion. Discovery of a connection between LeuRS and GLUT1, as well as LeuRS and SREBP-1c, open a new chapter in our understanding of the molecular mechanism for regulating lactation. Taken together with our results presented here, we propose that LeuRS regulates cell proliferation and lactation in DCMECs by sensing intracellular leucine concentration.

## 3. Experimental Section

### 3.1. Cell Preparation and Treatments

Reagents were purchased from Sigma-Aldrich Canada (Oakville, ON, Canada) unless otherwise stated. Fetal bovine serum (FBS) and Dulbecco modified Eagle medium: F12 (DMEM/F12) base were obtained from GIBCO BRL (Life Technologies, Carlsbad, CA, USA). Procedures involving animals (*n* = 3 per group) were approved by the Animal Care Committee of the Northeast Agricultural University.

Mammary gland parenchymal tissues were individually isolated from three mid-lactation Holstein dairy cows of high genetic merit. The tissue soaked with 75% ethanol for 5 min before removing a portion of the outer periphery of interstitial tissue and fascia, adipose tissue, *etc.* Organs were washed repeatedly with saline until the flow of liquid was clear. The parenchymal of tissues were sheared into 1 mm^3^ pieces, and these pieces were moved into the bottom of the cell culture bottles, about 30–40 pieces in one bottle. Then the cell culture bottles were placed at 37 °C, 5% CO_2_ for cell culture. After about 3–4 h, 2 mL of culture medium was gently added into the culture bottles. The culture medium was changed on the third day and observed with an inverted microscope (DFC280, Leica, Germany) to assess whether cells arose from these pieces. The medium was changed once after day 3. For 15–30 days, the cell culture bottle was covered with fibroblasts, myoepithelial cells, mammary epithelial cells, *etc.* Dairy cow mammary epithelial cells were purified according to previous published reports [48]. Due to their sensitivity to digestion with 0.25% trypsin plus 0.02% EDTA, a nearly pure sample of DCMECs was isolated after three to four passages. Therefore, DCMECs passaged three to four times were used for experimental assays.

The purified cells were cultured in basic culture medium (DMEM/F12 base with 10% FBS added, 5 μg/mL insulin, 1 μg/mL hydrocortisone, 10 ng/mL epidermal growth factor, 100 U/mL penicillin and 0.1 mg/mL streptomycin). Prior to experimental treatments, cells were plated at 3 × 10^4^ cells/cm^2^ in six-well culture plates, and the medium was replaced with experimental culture medium (based on the basic culture mediun plus 5 μg/mL prolactin and a certain concentration of leucine). DMEM/F12 base media contained leucine (0.45 mmol/L) at a level sufficient for normal cellular processes, but not at a concentration optimal for DCMECs differentiation and lactation. Therefore, it was necessary for the addition of leucine to measure the dosage effects of leucine on lactation in DCMECs.

### 3.2. Cell Viability and Cell Proliferation Assay

Cell viability and cell proliferation were determined by using a CASY-TT Analyser System (Schärfe System GmbH, Reutlingen, Germany) according to the manufacturer’s instructions. After calibration with live and dead DCMECs, cursor positions were set to 11.75 to 50.00 μm (evaluation cursor) and 7.63 to 50.00 μm (standardization cursor). DCMECs were trypsinized and then diluted (1:100) with CASY electrolyte solution prior to examination. Three 100 μL aliquots were analyzed in sample [49].

### 3.3. Oil Red O Staining

Cells cultured in six-well culture plates were rinsed three times in phosphate-buffered saline (PBS), fixed in 4% (*w*/*v*) paraformaldehyde for 40 min, and then rinsed again with PBS. The lipid droplets in the cells were stained with 5% Oil red O in isopropanol for 15 min and then rinsed again with PBS. Nuclei were stained with Giemsa working solution for 15 min and then examined microscopically. The lipid droplets were stained red while nuclei were stained blue [22].

### 3.4. Analysis of Cell Cycle Progression by Flow Cytometry

The proportion of DCMECs in various different cell cycle phases was measured by flow cytometric analysis. DCMECs were plated at 3 × 10^4^ cells/cm^2^ and incubated in six-well culture plates with experiment culture medium without FBS for at least 36 h to synchronize the cell cycle. Then DCMECs were divided into groups as shown above. After 24 h, the cells were collected and washed twice with PBS. Each group was fixed overnight in ice-cold 70% ethanol at 4 °C, then rinsed three times in PBS and re-suspended in PBS containing 0.1 mg/mL RNase A and 5 μg/mL PI. After incubation for 30 min at room temperature in the dark, the cells were analyzed by flow cytometry using a FACS Calibur (BDFACS AriaTM Cell Sorter 334078, Becton-Dickinson, San Jose, CA, USA). Analysis of cell cycle distribution was subsequently performed using the Modfit software [50].

### 3.5. Small Interfering RNA Transfection

DCMECs were transfected with either LeuRS-specific siRNA (transfection LeuRS siRNA, GenePharma Co., Ltd., Shanghai, China) or negative control siRNA (negative control RNA-oligonucleotides, GenePharma Co., Ltd., Shanghai, China), using Lipofectamine™ 2000 (LF2000, Invitrogen, Camarillo, CA, USA) according to the manufacturer’s protocol. After about 4–6 h, the medium in the three groups was replaced with experimental culture medium and contained an extra 1.2 mmol/L leucine. The sequences of siRNA were listed below. LeuRS-specific siRNA: sense5′-GCAGGUCCUGUGGAUGAAATT-3′, antisense5′-UUUCAUCCACAGGACCUGCTT-3′; negative control siRNA: sense5′-UUCUUCGAACGUGUCACGUTT-3′, antisense5′-ACGUGACAC GUUCGGAGAATT-3′.

### 3.6. Immunohistochemistry

Slides and coverslips were soaked overnight in chromic acid solution. Then, the slides were rinsed with tap water to remove residual acid, rinsed with distilled water three times, and then rinsed with 75% ethanol. Before use, the frozen sectioning machine (CM 1850, Leica, Wetzlar, Germany) was pre-cooled to −20 °C for 2 h, and then the slices were made into 7–8 μm. The labeled frozen sections were fixed in 4% (*w*/*v*) ice-cold paraformaldehyde for 10 min, and then rinsed in PBS at room temperature for 5 min. Next, fixed tissues were incubated in blocking buffer (phosphate-buffered saline with 5% BSA and 0.1% TritonX-100 (Solarbio, Co., Ltd., Shanghai, China)) for 1 h at 37 °C, before incubation with anti-LeuRS primary antibody (Santa Cruz Biotechnology Inc., Santa Cruz, CA, USA) at 1:100 dilution for 1.5 h at 37 °C. After rinsing in PBS/T, specimens were incubated in the dark with FITC-conjugated donkey anti-rabbit IgG at a 1:200 dilution for 1 h at 37 °C. Then the slides were incubated in blocking buffer again, incubated with anti-Giantin primary antibody (Santa Cruz Biotechnology Inc., Santa Cruz, CA, USA) at 1:100 dilution for 1.5 h at 37 °C. Specimens were incubated in the dark with Cy5-conjugated donkey anti-goat IgG at 1:200 dilution for 1 h at 37 °C, and afterwards rinsed three times in PBS/T, then incubated with DAPI for 15 min at 37 °C, and rinsed in PBS/T; the coverslips were visualized using a Leica TCS-SP2 AOBS confocal laser scanning microscope [51].

### 3.7. Immunofluorescence

DCMECs were seeded on glass coverslips to 30%–50% confluency in six-well plates. The cells were rinsed twice with PBS and fixed in 4% (*w*/*v*) ice-cold formaldehyde at 4 °C for 10 min. The slides were rinsed three times with TBS/T for 5 min. To detect endogenous CK18, β-Casein and LeuRS, fixed DCMECs were incubated in blocking buffer (Tris-buffered saline with 5% BSA and 0.1% TritonX-100) for 1 h at 37 °C, and then incubated with anti-CK18 primary antibody (Santa Cruz Biotechnology Inc., Santa Cruz, CA, USA), anti-β-Casein primary antibody (Abbiotec, San Diego, CA, USA) and anti-LeuRS primary antibody at 1:100 dilution for 1.5 h at 37 °C, respectively. After rinsing three times in TBS/T, specimens were incubated in the dark with FITC-conjugated second antibodies at 1:200 dilution for 1 h at 37 °C and incubated with PI or DAPI for 15 min at 37 °C, respectively. Finally, after rinsing three times in TBS/T, the coverslips were visualized using a Leica TCS-SP2 AOBS confocal laser scanning microscope [52].

### 3.8. RNA Extraction and Quantitative Real-Time PCR

Total RNA extraction of DCMECs and analysis method were used according to the previous method of our laboratory reported by Lu *et al*. [53,54]. The total RNA was reverse transcribed into cDNA using primescript reverse transcriptase (TaKaRa, Dalian, China) according to the manufacturer’s protocol. qRT-PCR reactions were performed using real-time PCR Kit Sensimix™ SYBR&Flurescen, and the analysis was performed by an ABI PRISM 7300 RT-PCR System (Applied Biosystems, Foster City, CA, USA) in a total volume of 25 μL using 96-microwell plates. At least three wells were used for each gene in an independent experiment, and all of the following gene mRNAs were normalized to β-Actin mRNA level. The primers of these gene transcripts were as follows: LeuRS: sense5′-TCTGGAGAAGATGGGACCTCA-3′; antisence5′-GCTCAAGAGAGTTGGTCAGGTAGACT-3′; mTOR: sense5′-ATGCTGTCCCTGGTCCTTATG-3′, antisense5′-GGGTCAGAGAGTGGCCTT CAA-3′; S6K1: sense5′-CTGGGTGAAGAATGGAAGGG-3′, antisense5′-CGAACTCTGCCATG GGTCA-3′; β-Casein: sense5′-AACAGCCTCCCACAAAAC-3′, antisense5′-AGCCATAGCCTC CTTCAC-3′; SREBP-1c: sense5′-AGTAGCAGCGGTGGAAGT-3′, antisense5′-GCAGCGGCTCT GGATT-3′; Cyclin D1: sense5′-CCGTCCATGCGGAAGATC-3′, antisense5′-CAGGAAGCGGTCC AGGTAG-3′; GLUT1: sense5′-CTTCATCCCAGCCCTGTT-3′, antisense5′-ACCTTCTTCTCC CGCATC-3′; β-Actin: sense5′-AAGGACCTCTACGCCAACACG-3′, antisense5′-TTTGCGGTGGA CGATGGAG-3′. Data analysis was performed by the comparative *C*_t_ method by using the SDS V 1.2 software.

### 3.9. Western Blotting Analysis

Western blotting analysis was performed using standard techniques reported by Huang [55]. Total cell lysate containing about protein 30 μg was separated on a 10% SDS-PAGE gel and transferred onto nitrocellulose membranes (Bio-RAD Company, Hercules, CA, USA). Membranes were blocked in 5% skim milk (in Tris-buffered saline with 5% skim milk and 0.1% Tween-20). Membranes were probed with primary antibodies specific for the following antibodies: LeuRS, GLUT1, Cyclin D1, SREBP-1c (the mature form, 68 kD) (Santa Cruz Biotechnology Inc., Santa Cruz, CA, USA); p-mTOR, mTOR, p-S6K1, S6K1, (Cell Signaling Technology, Beverly, MA, USA); β-Casein (Abbiotec, San Diego, CA, USA) and β-Actin (Santa Cruz, CA, USA), followed by a second incubation with secondary antibodies (1:1000) conjugated to HRP (ZSGB-BIO, Beijing, China). The chemiluminescence detection of HRP-conjugated secondary antibodies was performed using Super ECL plus (ApplyGEN, Beijing, China). Analysis of these western blotting results were subsequently performed using the Quantity One software.

### 3.10. Quantitation of Secreted Triglyceride and Lactose in the Culture Medium

The amount of triglyceride and lactose secreted into the culture medium was determined using commercially available assay kits (triglyceride detection kit: ApplyGEN, Beijing, China; lactose assay kit: Lactose/d-galactose (Rapid) Assay Kit, Megazyme, Ireland, UK) according to the manufacturer’s recommended protocol [56].

### 3.11. Statistical Analysis

All data were expressed as the mean ± standard deviation (*n* = 3) and were tested for statistical significance (*p* < 0.05 or *p* < 0.01) by using SPSS 16.0 (SPSS Inc., Chicago, IL, USA). A statistical comparison of the means among the groups was performed using one-way analysis of variance. Differences between the means of individual groups were analyzed by Tukey *post hoc* tests. Statistical significance was declared at *p* < 0.05 or *p* < 0.01.

## 4. Conclusions

Leucine can modulate cell proliferation and lactoprotein, as well as lactose and triglyceride synthesis, by increasing expression of LeuRS in DCMECs. LeuRS is a key mediator for amino acid signaling to mTORC1 that functions in response to changes in the intracellular leucine concentration. LeuRS can mediate cell proliferation and the expression of mTOR, S6K1, SREBP-1c, GLUT1, Cyclin D1, and β-Casein to promote lactation in DCMECs.
